# Identification of methionine -rich insoluble proteins in the shell of the pearl oyster, *Pinctada fucata*

**DOI:** 10.1038/s41598-020-75444-4

**Published:** 2020-10-27

**Authors:** Hiroyuki Kintsu, Ryo Nishimura, Lumi Negishi, Isao Kuriyama, Yasushi Tsuchihashi, Lingxiao Zhu, Koji Nagata, Michio Suzuki

**Affiliations:** 1 Department of Applied Biological Chemistry, Graduate School of Agricultural and Life Sciences, The University of Tokyo, 1-1-1 Yayoi, Bunkyo-ku, Tokyo, 113-8657 Japan; 2 Center for Health and Environmental Risk Research, National Institute for Environmental Studies, 16-2 Onogawa, Tsukuba-city, Ibaraki, 305-8506 Japan; 3 Institute for Quantitative Biosciences, The University of Tokyo, 1-1-1 Yayoi, Bunkyo-ku, Tokyo, 113-8657 Japan; 4 Mie Prefecture Fisheries Research Institute, 3564-3 Hamajima, Hamajima-cho, Shima-city, Mie 517-0404 Japan

**Keywords:** Biogeochemistry, Ocean sciences

## Abstract

The molluscan shell is a biomineral that comprises calcium carbonate and organic matrices controlling the crystal growth of calcium carbonate. The main components of organic matrices are insoluble chitin and proteins. Various kinds of proteins have been identified by solubilizing them with reagents, such as acid or detergent. However, insoluble proteins remained due to the formation of a solid complex with chitin. Herein, we identified these proteins from the nacreous layer, prismatic layer, and hinge ligament of *Pinctada fucata* using mercaptoethanol and trypsin. Most identified proteins contained a methionine-rich region in common. We focused on one of these proteins, NU-5, to examine the function in shell formation. Gene expression analysis of NU-5 showed that NU-5 was highly expressed in the mantle, and a knockdown of NU-5 prevented the formation of aragonite tablets in the nacre, which suggested that NU-5 was required for nacre formation. Dynamic light scattering and circular dichroism revealed that recombinant NU-5 had aggregation activity and changed its secondary structure in the presence of calcium ions. These findings suggest that insoluble proteins containing methionine-rich regions may be important for scaffold formation, which is an initial stage of biomineral formation.

## Introduction

Molluscan shells are biominerals, mineralized hard tissue, mainly composed of calcium carbonate and small amounts of organic matrices. Shells have distinctive microstructures formed by various organic matrices controlling calcium carbonate crystallization^1^. The organic–inorganic composites in biominerals have unique features, such as lamellar structures or well-oriented crystals, which lead to superior hardness and stiffness of the composite compared to geological minerals^2^. Many researchers have studied their mechanism of formation for industrial applications.

**Figure 1:**
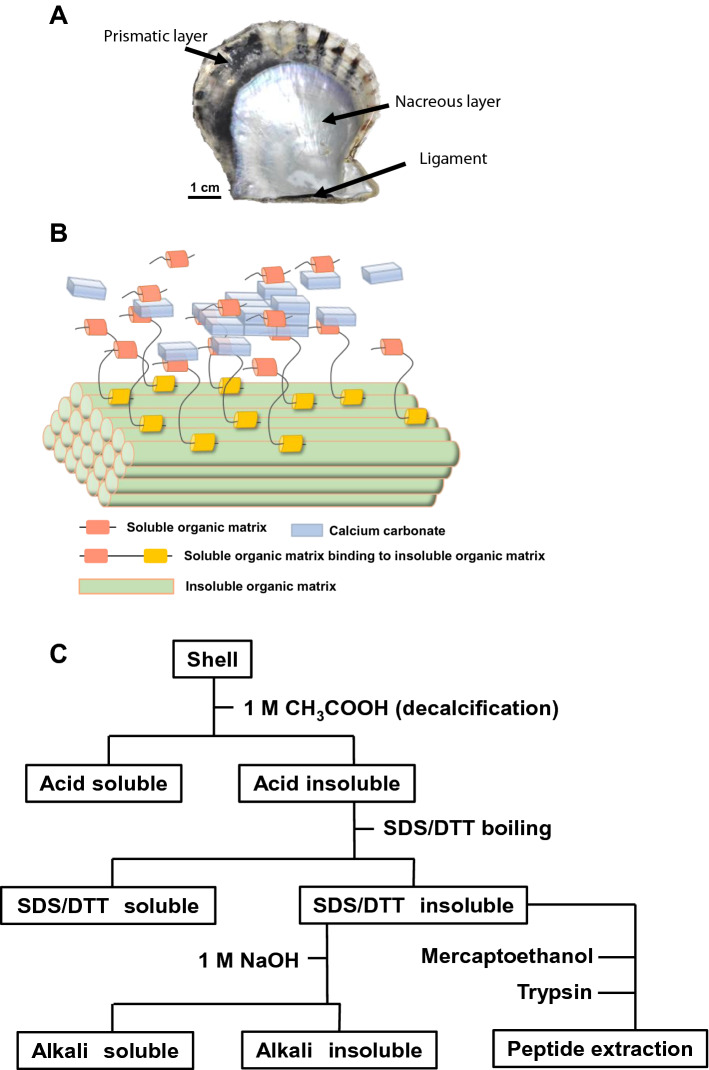
(**A**) Shell structure of *Pinctada fucata*. (**B**) A model for a hierarchical structure of organic matrices. The insoluble organic matrices containing chitin become the scaffold. A nucleation of calcium carbonate occurs around the insoluble organic matrices mediated by the soluble organic matrices which have chitin binding domain (yellow) and calcium carbonate-interaction domain such as acid-rich domain (pink). Other soluble organic matrices control the crystal growth. (**C**) A flow chart for the extraction of organic matrices.

*Pinctada fucata* is a bivalve known as the Japanese pearl oyster. The shells have three mineralized structures: the nacreous layer, prismatic layer, and a hinge ligament (Fig. 1A). The nacreous layer, an inner layer of the shell, has a lamellar structure formed by thin polygonal aragonite tablets and thin organic membrane stacking^3,4^. The aragonite tablets are approximately 300 nm thick, approximately 5 µm in diameter, are arranged horizontally between organic membranes, and are stacked vertically. The prismatic layer, an outer layer of the shell, has a honeycomb structure composed of calcite prisms^5,6^. The calcite prisms range from 20–40 µm in diameter and are approximately 120 µm in length. Each calcite prism is surrounded by an organic framework. The hinge ligament, located in the hinge of the valve, has extremely thin fibrous aragonites of approximately 50 nm in thickness^7,8^. The fibrous aragonites are densely bunched, and fibrous aragonite fibres are surrounded by dense organic matrices. *P. fucata* generates these different microstructures of calcium carbonate in the shell by secreting specific organic matrices, such as chitin and proteins in each place. In general, according to their solubility, organic matrices can be classified into three groups: an acid (water or ethylenediaminetetraacetic acid (EDTA))-soluble fraction, an acid-insoluble/detergent-soluble fraction, and an acid-insoluble/detergent-insoluble fraction^9^ (Fig. 1B). The main function of acid-insoluble organic matrices is to provide a scaffold on which crystal nucleation and crystal growth occur. The acid-soluble and acid-insoluble/detergent-soluble organic matrices facilitate crystal nucleation and control crystal growth, including crystal polymorphism, morphology, and orientation. In previous studies, to extract organic matrices, the shell has been first decalcified with acid (normally acetic acid) or EDTA. At this point, the proteins that exist freely and have high solubility are easily extracted by acid or EDTA. The remaining insoluble organic matrices are a complex of chitin and proteins that bind to chitin with specific domains, such as a chitin-binding domain. To extract components from these complexes by denaturing the proteins, sodium dodecyl sulfate (SDS) or urea have been mainly used, and acid-insoluble/detergent-soluble organic matrices have been obtained. Almost all proteins that have been identified to date are derived from the acid-soluble fraction or acid-insoluble/detergent-soluble fraction: nacrein^10^, MSI60^11^, Pif^12^, and N16^13,14^ in the nacreous layer, prismalin-14^15^, prismin^16^, and MSI31^11^ in the prismatic layer, and LICP^17^ and TIMP^18^ in the ligament. However, large amounts of unknown organic matrices remained in the complex after those treatments, indicating that the remaining proteins make strong cross-links to chitin or each other to strengthen the scaffold. Thus, a new approach is required to analyse these proteins.

Recently, our research group succeeded in identifying a novel protein, LMP, from a strongly cross-linked structure in the acid-insoluble/detergent-insoluble fraction of the ligament using a combination of mercaptoethanol and trypsin^7^. Mercaptoethanol is known as a reducing agent that reduces and cleaves the disulphide bond between cysteines to denature proteins^19^. Trypsin is a protease that recognizes specific amino acids, such as lysine and arginine, and cleaves proteins into peptides^20^. Mercaptoethanol is expected to loosen the strongly cross-linked proteins, even if only slightly, for trypsin to enzymatically act on those proteins. Although it was difficult to completely extract the cross-linked proteins, this problem was solved by obtaining the peptide fragments from cross-linked proteins and deducing the whole amino acid sequence of proteins from those fragments using the genome data of *P. fucata*. In this study, we extracted and identified insoluble proteins from the nacreous layer, prismatic layer, and the ligament. Furthermore, using the recombinant protein prepared in reference to the obtained sequence, the function of the protein on shell formation was examined.

## Results

### Measurement of the proportion of chitin and proteins in organic matrices in the shell microstructure

To investigate the proportion of calcium carbonate, insoluble protein, and chitin in the microstructures of *P. fucata* shells, the shell was separated into nacreous, prismatic, and ligament microstructures and each was treated with acid, subsequently detergent (a mixture of SDS and DTT), and alkali reagents. Figure 1C shows the scheme of treatments. We obtained the weight of each soluble and insoluble components. The percentages of these components were shown in Table 1. The detergent-soluble and -insoluble fraction were not described in Table 1, because the detergent-soluble organic matrices comprised less than the detection limit in all microstructures. The acid-soluble fraction was composed of calcium carbonate and soluble proteins. In the acid-insoluble fraction which is mainly the complex of chitin and proteins, the weight of chitin can be obtained as the alkali insoluble material since due to the strong hydrogen bonding, chitin is hardly dissolved with 1 M sodium hydroxide and boiling^21^, which dissolves the proteins cross-linking with chitin or each other. In the nacreous microstructure, the acid-insoluble organic matrices comprised approximately 4% (w/w), while the alkali-soluble proteins comprised 74%, and alkali-insoluble chitin comprised 26%. In the prismatic microstructure, the acid-insoluble organic matrices comprised 9% (w/w), while the alkali-soluble proteins comprised 86%, and alkali-insoluble chitin comprised 14%. The ligament microstructure contained a large amount of acid-insoluble organic matrices (38%). However, alkali-insoluble chitin did not exist in the ligament because alkali treatment with boiling completely dissolved the acid-insoluble organic matrices. From these data, acid-insoluble (and detergent-insoluble) proteins binding to chitin (although chitin did not exist in the ligament) were the main components that compose the scaffold of organic matrices in these microstructures. These proteins make such a strong complex that SDS and DTT cannot denature it. Therefore, to identify these insoluble proteins, we used trypsin digestion on the solid material of acid-insoluble/detergent-insoluble fraction, to obtain scaffold protein peptide fragments that formed a complex with chitin.

**Table 1:** Proportion of the components in the shell of *P. fucata.*

Sites	Acid soluble fraction (%)^1^	Acid insoluble fraction (%)^2^	Acid insoluble fraction	
			Alkali soluble fraction (%)^3^	Alkali insoluble fraction (%)^4^
Nacreous layer	96	4	74	26
Prismatic layer	91	9	86	14
Ligament	62	38	100	0

^1^Calcium carbonate + proteins, ^2^ chitin + proteins, ^3^ proteins, ^4^ chitin.

### Identification of the insoluble protein

The acid-insoluble/detergent-insoluble organic matrices from the nacreous layer, prismatic layer and ligament were treated with mercaptoethanol and were digested by trypsin to extract peptides. After digestion, the supernatant of peptide solution was analysed by liquid chromatography-tandem mass spectrometry (LC–MS/MS). LC–MS/MS analysis data of the proteins with the two highest scores from each structure are shown in Supplementary Table S1-S3. We used the draft genome database of *P. fucata* for the identification of proteins.

**Figure 2:**
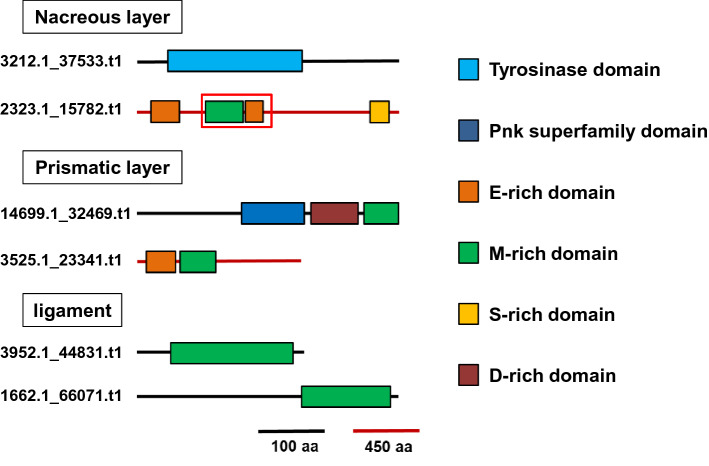
A schematic diagram of domain structures of proteins deduced from peptides extracted from the acid-insoluble/detergent insoluble fraction in the nacreous layer, prismatic layer and ligament. The number of gene ID is described at left side. A red color boxes the recombinant region we made.

From the data of the nacreous layer, basic local alignment search tool (BLAST) results revealed that the amino acid sequence of the highest score (pfu_aug1.0_3212.1_37533.t1) encoded tyrosinase of approximately 45 kDa (Fig. 2). Tyrosinase is known as an enzyme that catalyses the melamine synthesis, but some tyrosinases identified from bivalve shells may function in the intermolecular cross-linking^22^ The second highest score (pfu_aug1.0_2323.1_15782.t1) encoded nacre unknown protein-5 (NU-5) (Fig. 2), which has been reported from the nacreous layer in a previous shell proteome study^23^. NU-5 was approximately 180 kDa and had no homolog with known proteins from the BLAST search. Although NU-5 had no conserved domain and the function or characteristics have yet to be analysed, NU-5 had glutamic acid, methionine, and serine-rich regions and repeats, indicating the relation to a biomineral formation. Whole amino acid sequences are shown in Fig. S1.

From the prismatic layer, BLAST search results revealed that the protein with the highest score (pfu_aug1.0_14699.1_32469.t1) encoded prismatic, nacre unknown protein (P,NU-5) (Fig. 2), which has been reported from proteome analysis of the nacreous and prismatic microstructures^23^. P,NU-5 was approximately 45 kDa and had no homolog with known proteins from the BLAST search. P,NU-5 contained a conserved domain of the Pnk superfamily that included NADP phosphatase/NAD kinase bifunction, and methionine-rich and aspartic acid-rich regions. The protein with the second highest score (pfu_aug1.0_3525.1_23341.t1) encoded a novel protein with no homologous protein (Fig. 2). This novel protein was approximately 120 kDa. The protein also had a glutamic acid-rich region and a methionine-rich region containing unique glutamine repeats. Whole amino acid sequences are shown in Fig. S2.

In the ligament, the microstructure contains large amounts of acid-insoluble/detergent-insoluble proteins without chitin. BLAST search results revealed that the protein with the highest score (pfu_aug1.0_3952.1_44831.t1) encoded LMP (Fig. 2). LMP has been reported in previous research as a major components of insoluble organic matrices in ligament, instead of chitin in other structure, and contains abundant methionine residues^7^. The protein with the second highest score (pfu_aug1.0_1662.1_66071.t1) was a novel protein (Fig. 2). This protein was approximately 50 kDa. The protein had a methionine-rich region at the C-terminal domain. Whole amino acid sequences are shown in Fig. S3.

From the identification of insoluble protein sequences, proteins whose function in the shell has yet to be determined were obtained. Almost all proteins obtained from the nacreous, prismatic, and ligament microstructures had a methionine-rich region in common, indicating the key role in the scaffold formation of biominerals. Some proteins in the nacreous and prismatic layer had a glutamic acid-rich or aspartic acid-rich region that is common in biomineral proteins, whereas the proteins in the ligament had no acidic amino acid-rich region. This difference may lead to less amount of calcium carbonate in ligament and the specific function as hinge. For the next step, it is important to clarify the function of proteins containing the methionine-rich region on the scaffold formation. Herein, we firstly focused on NU-5 for the subsequently analyses, because the progress in studies on the nacre formation is more expected than on the prismatic and ligament formation, because of beautiful colour and commercial value. However, the formation mechanism of organic scaffold in the nacre is hardly unknown in spite of many reports about matrix proteins in the nacre.

### Gene expression analysis of NU-5

**Figure 3:**
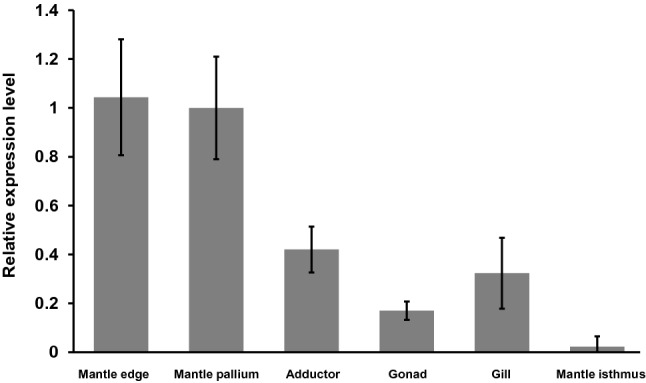
Relative gene expression levels of NU-5 in each tissue of *P. fucata*. Real-time quantitative PCR (qPCR) was performed to evaluate relative gene expression levels compared with the mantle pallium. The values are relative to that of the mantle pallium. Data are expressed as the mean ± S.E. (*n* = 3).

To analyse tissue-specific gene expression of NU-5, the expression levels of NU-5 in the mantle pallium, mantle edge, mantle isthmus, gill, adductor, and gonad were measured using quantitative real time-polymerase chain reaction (RT-PCR, Fig. 3). Expression levels of NU-5 in the mantle pallium and mantle edge were high, whereas expression levels in the adductor, gonad, and gill were low. Considering the mantle edge and pallium are the tissues secreting the components of the prism and nacre, the expression levels of NU-5 in the mantle edge and pallium were more than twice than those of other tissues suggesting that NU-5 was related to the shell formation. The small expression in the adductor, gonad and gill may imply the other function of NU-5. The mantle isthmus that secretes the ligament microstructure showed extremely low expression level of NU-5 indicating that NU-5 was not related to the ligament formation.

### Knockdown of NU-5 using RNA interference (RNAi)

**Figure 4:**
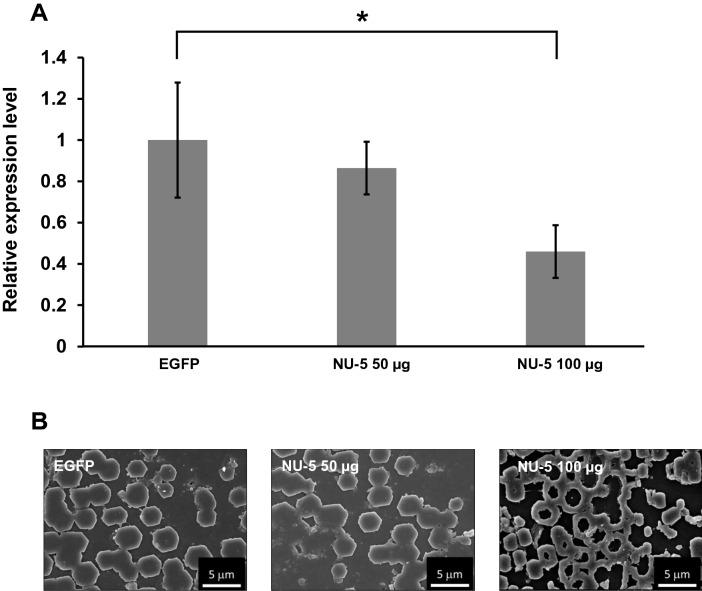
(**A**) Relative gene expression levels of NU-5 4 days after injection of dsRNAf of NU-5. dsRNA of EGFP was injected as negative control. qPCR was performed to evaluate relative gene expression levels compared with EGFP. Data are expressed as the mean ± S.E. Statistically significant differences were determined by t-test (*p < 0.05, *n* = 3). (**B**) Surface microstructure images of the nacreous layer 4 days after injection observed using SEM. The surface is the growth front of the nacre. In the condition of NU-5 100 µg, the normal nacre formation was disrupted.

To investigate the function of NU-5 in the nacreous layer, a knockdown experiment using RNAi was performed. The dsRNA targeting NU-5 were injected into a living *P. fucata*, which was then cultured in seawater for four days. Because *P. fucata* does not contain the green fluorescent protein (EGFP) gene, EGFP-specific dsRNA was also injected into living *P. fucata* as a negative control. After cultivation for four days, mRNA expression levels of NU-5 were measured using quantitative RT-PCR (Fig. 4A). We normalized the expression level of each injection by the expression level of negative control. The expression levels of individuals injected with NU-5-specific dsRNA at a concentration of 50 µg showed no significant difference compared to that of the negative control. However, in those injected with dsRNA of NU-5 at a concentration of 100 µg, expression levels decreased to approximately 50% compared to that of the negative control. The growth surface of the nacreous layer after injection was observed by scanning electron microscopy (Fig. 4B). The observed area was fixed at the region approximately 1 cm from the boundary between the nacreous and prismatic layers. In the nacreous layer of the individuals injected with EGFP-specific dsRNA and 50 µg NU-5-specific dsRNA, normal aragonite tablets were observed. Each tablet increased in size and bound each other to form the stacked structure of the nacreous layer. However, in individuals injected with 100 µg NU-5-specific dsRNA, almost all aragonite tablets exhibited abnormal structures with centre holes. The normal tablet grows from the centre to the outer layer of the tablet with a hexagonal or circular shape, and produces a flat surface. The whole tablet showed a strange shape and rough surface.

### Aggregation activity of recombinant NU-5 protein measured with dynamic light scattering (DLS)

To further examine the function of NU-5 in forming the organic scaffold in the nacreous layer, recombinant proteins, including the methionine-rich and glutamic acid-rich region (rMERP) of NU-5 were prepared. The sequence of the recombinant protein is shown in Fig. S4A. rMERP was expressed with a His-tag at the N-terminal domain after transformation (Fig. S4B). After the crude rMERP was extracted from *E. coli* cells and purified using a Ni column, a single rMERP (Fig. S4C).

**Figure 5:**
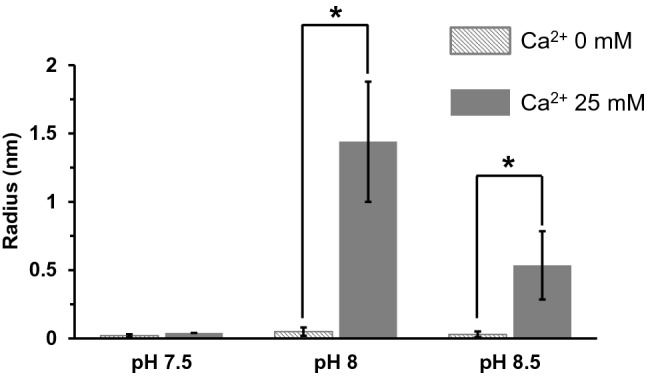
A particle size of the rMERP measured using dynamic light scattering (DLS). The aggregation activity of rMERP in the absence or presence of calcium ion at pH 7.5, 8.0 and 8.5 was measured. Data are expressed as the mean ± S.E. Statistically significant differences were determined by t-test (*p < 0.05, *n* = 3).

Since NU-5 had a strong cross-linked structure with chitin and proteins in the nacreous layer, rMERP was likely to have aggregation activity. Therefore, using DLS, we evaluated whether rMERP aggregated with or without calcium ions. DLS measured the distribution of particle size using the differences in the Brownian motion speed of the particle in solution. Figure 5 shows the particle size distribution of rMERP with or without calcium ions at pH 7.5, 8.0, and 8.5. The aggregation activity of rMERP without calcium ions was hardly detected, regardless of pH. However, aggregated large particles of rMERP with calcium ions at pH 8.0 were detected. Although a few particles were detected at pH 8.5, the particle size of pH 8.5 was approximately half of that at pH 8.0. The aggregation activity of rMERP at pH 7.5 was hardly detected, similar to the condition with no calcium ions.

### Analysis of steric structural changes in rMERP using Nile red staining and circular dichroism (CD)

DLS measurements demonstrated that rMERP aggregated particles the most with calcium ions at pH 8.0. Nile red staining was used to analyse the change in the steric structure of rMERP. When Nile red binds to a hydrophobic site in the protein, the fluorescence of the reagent will appear. However, the free state of Nile red does not show fluorescence because of quenching. When the steric structure of the protein was broken and Nile red bound to the exposed hydrophobic regions, the fluorescent spectra of the protein with Nile red changed. Measurement of fluorescence spectra for rMERP with Nile red showed a change in steric structure in the hydrophobic region of rMERP. The fluorescence intensity of rMERP with Nile red was measured at pH 7.5, 8.0, and 8.5, with or without calcium ions (Fig. S5). The fluorescence intensity of rMERP, with or without calcium ions, at pH 8.0 was the strongest. The intensity at pH 7.5 was the lowest. However, the difference of fluorescence intensity in each condition was very small. There were no significance differences by statistical analyses. These results complemented the DLS results, suggesting that the steric structure of rMERP was the most changed at pH 8.0.

DLS and Nile red staining indicated that a condition of pH 8.0 in the presence of calcium ions induced the formation of an rMERP aggregated form with a changed protein structure. To analyse the change in the detailed protein structure, the secondary structure change of rMERP was estimated using the CD spectrum. The CD spectrum plots the differences in the absorption rate between left-handed and right-handed circularly polarized light when the circular light passes through the optical isomer. In the case of proteins, the secondary structure was estimated by comparing the spectrum of the sample with that of a known protein whose secondary structure had previously been identified. Fig. S6 shows the results for the condition with or without calcium ions at pH 8.0. The anti-parallel, which was a sheet structure, had the highest percentage of all structures of rMERP in the absence of calcium ions (Table 2). The helix was much lower than the other secondary structures. On the other hand, content of helix structure increased in the presence of calcium ions. The percentage of the anti-parallel structure decreased by the increase of helix percentage.

**Table 2:** Estimated secondary structure content (%).

	No Ca^2+^	Ca^2+^
Helix	1.8	16.7
Anti-parallel	49.3	34.9
Parallel	4.0	2.5
Turn	15.7	12.1
Others	29.3	33.8

## Discussion

Previous studies have mainly analysed acid- or EDTA-soluble organic matrix in the shell, whereas an insoluble organic matrix has not been particularly studied because of the extraction difficulty. Although some researchers have identified insoluble organic matrices using denaturants, many residual proteins remained after extraction, as shown in the results of the alkali-soluble fraction (Table 1). These proteins, which are strongly cross-linked with chitin, may be necessary for the self-assembly of the organic matrix to form the organic scaffold in the shell. However, it is difficult to analyse the detailed structure and function of these proteins. In previous reports, from the acid (or EDTA)-soluble fraction or denaturing regent (SDS, DTT and urea)-soluble fraction, the various proteins have been identified^24,25^. For example, nacrein^10^, pearlin^13,14^, N19^26,27^, Pif^12^ in the nacre, prismalin-14^15^, prismin^16^, shematrins^28^ in the prism and LICP^17^ in the ligament. These proteins were basically related to calcium carbonate crystallization such as a supply of bicarbonate ions and an interaction with calcium ions or calcium carbonate to induce a specific crystal polymorph and morphology. Chitinase has also been identified as acid-insoluble/detergent-soluble protein in the prism and the function on biomineralization is considered to facilitate to make chitin nanofiber which interacts more with calcium carbonate, or to facilitate to shell remodelling^29,30^. Although many biomineral proteins were reported, our result showed that the functions of most proteins obtained from the acid-insoluble/detergent-insoluble fraction in the nacreous, prismatic, and ligament microstructures using mercaptoethanol and trypsin digestion have not been clearly clarified. MSI31 and MSI60 have been identified from acid-insoluble fraction by using cyanogen bromide that cleaves protein into peptides, which is similar way to our study, but the author treated acid-insoluble fraction directly with cyanogen bromide without treatment with detergent regent^11^. Considering that MSI31 and MSI60 were not detected in our data, they may mainly exist in detergent-soluble fraction. Thus, these suggested that proteins we identified have a novel function on biomineralization, especially the scaffold formation.

Focusing on the amino acid sequence of proteins that we identified, interestingly, proteins which contained methionine-rich regions were obtained from all three microstructures in common. Although several proteins that contain a methionine-rich region have been previously reported^7,31–33^, the function of biomineralization has hardly been investigated. In our study, the fact that most proteins obtained from the strongly cross-linked insoluble material had a methionine-rich region enabled us to consider that methionine was involved in the formation of a strong cross-linked structure. The formation of cross-links by methionine has been reported in some kinds of proteins, such as collagen and human growth hormone; collagen is known to have a fibrous structure in vertebrates. Collagen IV in mammals that comprises the extracellular matrix contains an oxidized methionine, forming a cross-linked structure with lysine, which may strengthen the structure of the extracellular matrix^34^. Oxidization of methionine in human growth hormone increases hydrophobicity and changes the steric structure of the protein, which facilitates self-assembly^35^. In addition to the methionine-rich domain, prolines were also often detected in some methionine-rich regions. Such proline-rich repetitive sequences were also seen in collagen. Generally, collagen comprises prolines or hydroxyprolines at a high rate, approximately 20%, which contributes to the formation of a triple helix^36^. Taken together, the methionine-rich region of the proteins that we identified may form a fibrous structure like collagen in the shell, and lead to the self-assembly of the fibrous structure to form the organic insoluble scaffold. Regarding the fibrous structure like collagen network, VWA-containing proteins (VWAPs) were detected among some molluscs including *P. fucata* by proteome of shell matrix proteins and transcriptome of tissues^37^. The VWA domain is found in collagens as well and exhibits an adhesion function through protein–protein interaction^38^. Among 10 VWAPs detected in the shell proteome of *P. fucata*, 8 VWAPs detected specifically from the nacre had highly homologous VWA domain with that of collagens in vertebrates, indicating the potential relation to the scaffold formation. However, VWAPs in some molluscs including *P. fucata* have no triple helix repeat (THR), which is necessary for self-assembly of collagen through triple-helix promoting to form the fibrous structure^39^. Thus, VWAPs in *P. fucata* cannot induce the self-assembly but form the cross-linkage by VWA. Combined with the findings of methionine-rich protein, methionine-rich proteins may basically form the organic matrix scaffold with chitin and VWAPs, instead of collagen in vertebrate bone.

On the other hand, our results showed some differences among the matrix proteins in each microstructure. The insoluble fraction in the nacreous layer contained tyrosinase. Tyrosinase oxidized the tyrosine or catechol to make quinone. Quinone reacted with each other to make the covalent bonds between the molecules^39^. Such covalent networks increase the hardness of the materials, indicating that the insoluble fraction of the nacreous layer may be oxidized by tyrosinase. The insoluble fraction in the nacreous and prismatic layers contained the matrix proteins which have methionine and glutamine-rich regions, while the insoluble fraction in the ligament did not contain. High content of glutamine increases the strength of hydrogen bond between molecules and gives the resistance against proteinase digestion^40,41^. As the nacreous and prismatic layers are the main shell structure, the insoluble materials may protect from the external invasion. The insoluble fraction in the ligament microstructure contained the highly repeated sequence with low complexity. Such extreme amino acid components may be related to the flexibility of molecules to increase the pressure resistance to open and close the bivalve shells. Although there were some different proteins from our result, which are likely to exhibit a specific function, we considered that the further analyses for function of methionine-rich protein found in common should be done. In this study, we focused on NU-5 obtained from the nacreous layer because NU-5 had both methionine-rich and acidic amino acid-rich regions.

The NU-5 knockdown experiment showed that the individuals injected with EGFP-specific dsRNA as a control showed normal aragonite tablets, while the individuals injected with NU-5-specific dsRNA showed abnormal aragonite tablets with a hole in the centre. This is probably because the self-assembly of the organic scaffolds between the tablets was disordered. Regarding the mechanism of formation of the laminate structure in the nacreous microstructure, the initial nacre is formed inside the dimples on the organic matrices covering the surface of the prismatic column^42^. Inside one dimple, horn-like aragonites nucleate and grow with random crystal orientation, which finally fill the hole to form a hemispheric dome. The domes grow concentrically and coalesce with each other to become the initial nacreous microstructure. The *c*-axis of the domes is preferentially oriented perpendicular to the surface by geometrical selection. In parallel with dome growth, lamellar thin organic sheets composed of chitin and proteins, such as NU-5, are formed on the domes. Subsequently, calcium carbonate is pushed from where aragonites meet each other to the upper layer through the narrow gap of the organic sheet, which becomes the nucleus of the upper crystals, termed the mineral bridge^43^. Each upper nucleus grows into a thin aragonite tablet along with an organic sheet. A previous study has reported that when Pif, the matrix protein associated with the aragonite formation of the nacreous microstructure in *P. fucata*, is knocked down, the lamellar structure becomes disordered because of the early formation of the tablets on the upper layer before the tablets on the lower layer completely form^24^. Knockdown of Pif decreased the secretion of Pif. Chitin could not form the normal organic sheets and maintain the lamellar structure, resulting in the random deposition of calcium carbonate. In this study, knockdown of NU-5 also induced abnormal tablets that had a hole in the centre, indicating that the organic sheet on the upper layer of the tablet may not be formed due to the functional disorder of self-assembly of organic matrices caused by the inhibition of NU-5. In the absence of an organic scaffold, calcium carbonates grew along the outer edge of the lower tablets. In addition, it is difficult for calcium carbonate to grow into thin crystals without the organic sheet. Therefore, NU-5 may play an important role in the self-assembly of organic scaffolds.

We prepared a recombinant protein containing a methionine-rich and glutamic acid-rich region from NU-5 (rMERP) to determine its function in the formation of the scaffold. The aggregation activity of rMERP was measured under different conditions. rMERP showed the most changed steric structure and was the most aggregated at pH 8.0, in the presence of calcium ions. This condition is close to sea water and the body fluid of *P. fucata*, suggesting that NU-5 has the capability of aggregation in vivo. In general, biomineral proteins, especially acidic proteins that have specific acidic amino acid-rich regions, possess high aggregated activity in the presence of calcium ions. The anion of calcium ions gathering around the cation of the acidic amino acid interrupts the electrostatic interaction between molecules^44^. Thus, the aggregation of rMERP may be mainly caused by glutamic acid. In the CD measurements, the percentage of helix structure significantly increased in the presence of calcium ions. The increase in helix may be due to aggregation because intramolecular or intermolecular hydrogen bonds occur at close distances, leading to helix formation. Similarly, it has been reported that Pif is also the most aggregated at pH 8.0, in the presence of calcium ions^45^. These results suggested that the multiple proteins in the organic matrix may synergistically work with each other to facilitate self-assembly at pH 8.0, in the presence of calcium ions.

**Figure 6:**
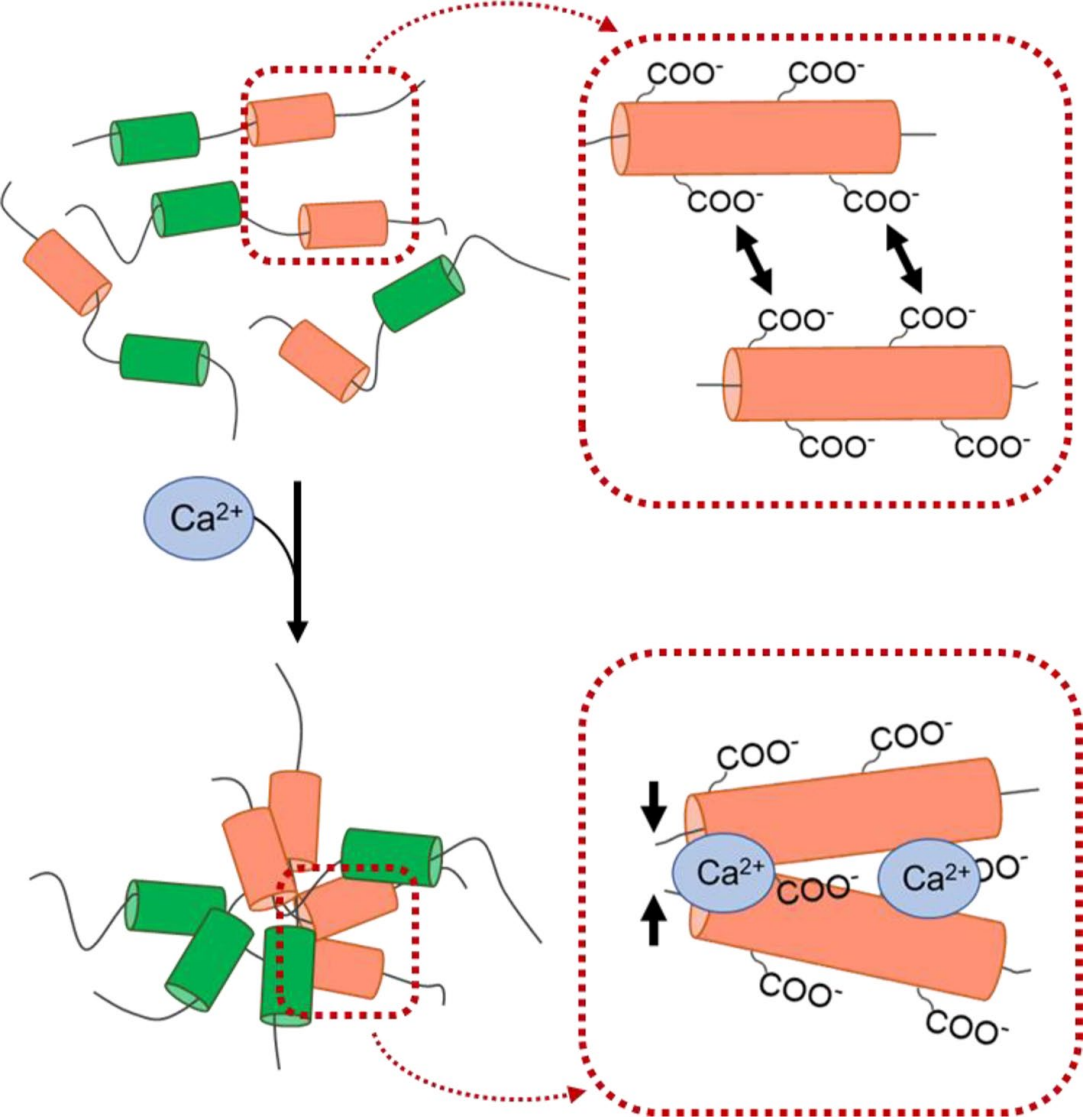
A schematic illustration for the proteins aggregation with calcium ion. Without calcium ion, proteins including acidic amino acid-rich region (pink) are dispersed to some extent because of an electrical repulsion between carboxy groups of acidic amino acids. When calcium ion comes, the cation of calcium ion disturbs the electrical repulsion, leading to the aggregation. After aggregation, the cross-linkage may occur by methionine-rich regions (green).

Figure 6 illustrates the schematic diagram for the function of NU-5. NU-5 has both a methionine-rich region and two glutamic acid-rich regions. As rMERP was aggregated in the presence of calcium ions via glutamic acid, NU-5 may have the capability of spontaneous aggregation in the conditions of calcification, the first step of self-assembly. In the next step, because the distance between proteins is reduced by aggregation, a cross-link may be formed among proteins, leading to formation of the organic insoluble scaffold. Although this schematic diagram illustrates only one kind of protein action, the various kinds of proteins in biomineralization would follow in this way to form the various insoluble organic scaffolds cross-linked with each other. Therefore, a more detailed analysis of the specific function of methionine for scaffold formation will be necessary. In this study, we successfully extracted and identified a peptide from solid insoluble organic materials that had been difficult to analyse using conventional methods. Additionally, proteins associated with biomineralization are generally easily aggregated, which leads to a difficulty in preparation of the recombinant in the soluble fraction after extraction from *E. coli*; however, the preparation of the recombinant methionine-rich region of NU-5 was successful. The results from this study provide the basis for further studies on the self-assembly mechanism of the organic matrix in biominerals.

## Materials and methods

### Measurement of the shell components

We used *Pinctada fucata* at age of three. *P. fucata* was cultured at the Ago bay, Mie prefecture, Japan by the Fishers Research Division, Mie Prefectural Science and Technology Promotion Center, and sent to The University of Tokyo, where *P. fucata* was cultured in natural sea water for 1–2 weeks. The shell was collected by removing soft bodies and washed with water and air-dried. The shell was separated into the nacreous layer, prismatic layer and ligament and each dry weight was measured. Each part was completely decalcified with 1 M acetic acid. After centrifugation, the precipitate was collected as the acid-insoluble material and washed with water, followed by freeze drying and the dry weight was measured. Then, the acid-insoluble material was boiled in 1% sodium dodecyl sulfate (SDS)/10 mL dithiothreitol (DTT)/50 mM Tris–HCl (pH 8.0) solution for 1 h and the dry weigh of the detergent-insoluble material was measured in the same way. The detergent-insoluble material was treated with 1 M sodium hydroxide at 100 °C for 12 h and washed with water, followed by the treatment with 1 M hydrochloric acid at room temperature for 5 min. This alkali and acid treatments were repeated at two more times. The alkali-insoluble material was freeze-dried and weighed. By comparing the weight before and after each treatment, the weight of each reagent-soluble organic matrix was obtained.

### Extraction and identification of proteins

After each part of shell was decalcified with acetic acid, the insoluble material was washed with water and boiled in 1% SDS/10 mL DTT/50 mM Tris–HCl (pH 8.0) solution for 10 min to obtain the detergent-insoluble material. The detergent-insoluble material was washed with water and treated in 100 µL 2-mercaptoethanol/10 mL Tris–HCl (pH 8.0) solution for reduction at 95 °C for 1 h. Then, the insoluble material was washed with water and treated in 200 µL 1 µg/µL trypsin (Promega)/5 mL 100 mM ammonium bicarbonate solution at 37 °C for 20 h. After centrifugation, the supernatant was collected as peptide extract to a new tube. This supernatant was completely dried and subsequently dissolved in 40 µL 0.1% trifluoro acetic acid (TFA)/2% acetonitrile solution, followed by application to LC–MS/MS (Thermo Fisher, Orbitrap Velos). The data from the LC–MS/MS was analyzed using the soft of Proteome Discover 2.1 and genome database of Pfu_aug1.0^46^.

### Gene expression analysis

Total RNA was extracted from the mantle edge, mantle pallium, adductor, gonad, gill and mantle isthmus, respectively, using Sepasol-RNA I Super G (Nacalai Tesque), following the manufacturer’s instructions. The cDNA was synthesized using PrimeScript RT reagent Kit (Perfect Real Time) (TaKaRa). 2 µL PrimeScript Buffer, 0.5 µL PrimeScript RT Enzyme Mix, 0.5 µL Oligo dT Primer (50 µM), 0.5 µL Random 6 mers (100 µM) and 1 µg total RNA in distilled water were mixed and RNase free water was added up to 10 µL. The samples were heated at 37 °C for 5 min for cDNA synthesis and at 85 °C for 5 s to stop the reaction, and stored at 4 °C. To quantity the gene expression levels, real-time quantitative PCR (qPCR) was performed using cDNA of each tissue and THUNDERBIRD Probe qPCR Mix (TaKaRa). Primers (NU-5_qPCR_F/NU-5_qPCR_R) were designed using the Primer3Plus software (Table 3)^47^. qPCR reaction mixture (20 µL) was adjusted according to the manufacturer’s instructions. qPCR was carried out on Applied Biosystems StepOne and StepOnePlus. Actin (amplified by primers: actin_qPCR_F/ actin_qPCR_R) (Table 3) was used as an internal reference for qPCR. To compare the expression levels of these genes, ΔΔCT method was used in the experiment^48,49^. Cycling parameters were following: 1 cycle of 10 min at 95 °C, 40 cycles of 15 s at 95 °C, 30 s at 60 °C and 30 s at 72 °C. Dissociation curves were analyzed at the end of each run to determine the purity of the product and specificity of amplification.

**Table 3:** Sequences of primers.

NU-5_qPCR_F	cctgagattgaaccacagca
NU-5_qPCR_R	caatagacatgccaccatcg
actin_qPCR_F	agagggaagcaaggatggat
actin_qPCR_R	cagaaggaaatcaccgcact
NU-5_RNAi_F	ggaccaggacctgatatggg
NU-5_RNAi_R	attccgcccatatcaggc
NU-5_RNAi_2ndF	taatacgactcactatagggggaccaggacctgatatggg
NU-5_RNAi_2ndR	taatacgactcactatagggattccgcccatatcaggc
NU-5_qPCR2_F	gacctgatgttggtggaatg
NU-5_qPCR2_R	ttcaggtcctagacccatgc

### RNA interference (RNAi)

To interference mRNA of NU-5, the dsRNA targeting NU-5 was prepared. The dsRNA of NU-5 was made by PCR with synthesized cDNA of mantle as template. 1st PCR was performed using Ex Taq (TaKaRa) and PCR reaction mixture (20 µL) was adjusted according to manufacturer’s instructions. Primers (NU-5_RNAi_F/ NU-5_RNAi_R) were designed to obtain amplicons of about 500 bp (Table 3). PCR cycling parameter was following: 1 cycle of 3 min at 96 °C, 35 cycles of 30 s at 96 °C, 30 s at 55 °C and 30 s at 72 °C. The samples were stored at 4 °C. The nest PCR was performed using 1st PCR product (100-fold diluted with distilled water) as template. Primers for nest PCR (NU-5_RNAi_2ndF/ NU-5_RNAi_2ndR) were designed to attach T7 promoter recognition sequence (Table 3). The nest PCR used the same conditions as 1st PCR, and the product was used as a template of making a dsRNA. In vitro Transcription T7 Kit (for siRNA Synthesis) (TaKaRa) was used to make dsRNA according to the manufacturer’s instructions. A dsRNA of enhanced green fluorescence protein (EGFP) was also prepared as a control. 30 µg of each dsRNA was dissolved with 50 µL PBS buffer and injected to adductor muscle of *P. fucata*. *P. fucata* was cultured for a day in natural sea water at 20 °C before the injection to reduce the stress. 4 days after injecting, to quantify the expression level of NU-5 in the mantle, qPCR was performed using the method described in the previous section. Sequences of used primers (NU-5_qPCR2_F/ NU-5_qPCR2_R) were shown in Table 3.

To observe the nacreous layer, soft bodies were removed from shells, and shells were washed with distilled water. Dried shells were attached on aluminum stage with carbon tape and coated with Pt–Pd. The surface structure of the nacre was observed using SEM S-4800 (Hitachi).

### rMERP preparation

A rMERP-inserted plasmid cloned into pET-28 vector with His-tag in N-terminal domain was sent by GenScript japan. *E. coli* competent cells BL21 (DE3) were transformed with the rMERP-insered plasmid, and transformants were cultured in LB broth containing 50 µg/mL kanamycin sulfate at 37 °C for 16 h for prior culture. One volume of the culture solution was added into new 100 volumes of LB broth containing 50 µg/mL kanamycin sulfate, followed by incubation at 37 °C. At an OD_600_ of 0.5, isopropyl-β-D-thiogalactopyranoside was added to be at 0.1 mM or 0.5 mM of final concentration and the culture solution was incubated at 25 °C for 24 h to induce the expression. After incubation, the culture solution was centrifuged to collect the precipitate and the precipitate was suspended into PBS to wash and remove LB broth. The bacterial cells suspended in PBS were disrupted by sonication. After centrifuging the suspension, the supernatant was collected. An aliquot of the suspension was boiled in the same volume of denaturing buffer (250 mM Tris–HCl (pH 6.8)/40% glycerol/20% 2-mercaptoethanol/20% SDS/0.005% bromophenol blue (BPB)) and applied to SDS–polyacrylamide gel electrophoresis (SDS-PAGE, 15% gel). After electrophoresis, the gel was stained with coomassie brilliant blue (CBB) to confirm the expression of rMERP. To purify rMERP, NaCl was added into the suspension to be 0.5 M NaCl and the suspension was applied to Ni Sepharose 6 Fast Flow (GE Healthcare japan) according to the manufacturer’s instructions. A concentration of imidazole in elution buffer was increased from 20 to 500 mM in order to determine the optimal elution condition. An aliquot of the eluate was applied to SDS-PAGE as described above. The eluate was concentrated by ultrafiltration using Amicon Ultra-15 (Merch) and diluted with 50 mM Tris–HCl (pH 7.4) to replace the buffer. A concentration of rMERP was measured using spectrophotometer.

### Dynamic light scattering (DLS)

DLS was measured using Protein Solutions DynaPro99 (Wyatt Technology) according to the instructions of instrument. The samples of 1 mg/mL rMERP in 50 mM Tris–HCl buffer with or without 25 mM CaCl_2_ were prepared. pH of Tris–HCl buffers were 7.5, 8.0 and 8.5. The measurement parameters were as follows: temperature of 20 °C, wavelength of 8316 Å, time of 150 s, count of 20 times. The particle size was calculated using a software of Dynamics.

### Nile red staining

The sample of 1 mg/mL rMERP in 50 mM Tris–HCl buffer with or without 25 mM CaCl_2_ were prepared. pH of Tris–HCl buffers were 7.5, 8.0 and 8.5. 1 µM of Nile red (Funakoshi) was added into the sample solution, followed by measurement of fluorescent intensity (Excitation wavelength: 553 nm, Emission wavelength: 637 nm) using fluorometer (GE healthcare Typhoon 9400).

### Circular dichroism (CD)

CD spectra was measured using Circular Dichroism spectrometer JASCO-820 (JASCO). The samples of 0.4 mg/mL rMERP in 50 mM Tris–HCl (pH 8.0) with or without 25 mM CaCl_2_ were prepared. The measurement parameters were as follows: wavelength increment 1 nm, response time 4 s, scan speed of 20 nm/min.

## Supplementary information

Supplementary Information 1.

Supplementary Information 2.

Supplementary Information 3.

Supplementary Information 4.

## Data Availability

The datasets generated during and/or analysed during the current study are available from the corresponding author on reasonable request.
