# Supplementary material for: Identification of methionine -rich insoluble proteins in the shell of the pearl oyster, Pinctada fucata
Source: Sci Rep. 2020 Oct 27;10:18335. doi: 10.1038/s41598-020-75444-4 (PMC7591529; doi:10.1038/s41598-020-75444-4)
Supplement: Supplementary file 4 — Supplementary Information 4. [file 41598_2020_75444_MOESM4_ESM.pdf]

Supplementary Materials

Identification of methionine -rich insoluble proteins in the shell of the pearl oyster, *Pinctada fucata.*

Hiroyuki Kintsu[1,2#], Ryo Nishimura[1#], Lumi Negishi[3], Isao Kuriyama[4], Yasushi Tsuchihashi[4], Lingxiao Zhu[1], Koji Nagata[1], Michio Suzuki[1]*.

[1]Department of Applied Biological Chemistry, Graduate School of Agricultural and Life Sciences, The University of Tokyo, 1-1-1 Yayoi, Bunkyo-ku, Tokyo 113-8657, Japan.

[2]Center for Health and Environmental Risk Research, National Institute for Environmental Studies, 16-2 Onogawa, Tsukuba-city, Ibaraki 305-8506, Japan.

[3]Institute for Quantitative Biosciences, The University of Tokyo, 1-1-1 Yayoi, Bunkyo-ku, Tokyo 113-8657, Japan.

[4]Mie Prefecture Fisheries Research Institute, 3564-3 Hamajima, Hamajima-cho, Shima-city, Mie 517-0404, Japan.

#: Hiroyuki Kintsu and Ryo Nishimura contributed equally to this work.

*To whom correspondence should be addressed.

Tel: +81-3-5841-5156

E-mail: amichiwo@mail.ecc.u-tokyo.ac.jp

Figure S1

A

pfu_aug1.0_3212.1_37533.t1 (tyrosinase)

```
1   MKTDFMQMIPNVRIIGGQHVSYVSECKIELRCESRYDTIAGMHSFAAYRYAHLGPSFLGW
61  HRVYLIMYEEALQEIRSDVVLCYWDSTLDFLMPGSTQRFTVAFSADLFGNGRGNVINGAF
121 ANWQLPGGGTLRRDIARAQFPNPPPSLTRPGIVDLIATDPSITSHTQIVRGGTGFTDPDT
181 GRVHTWEQEHDNTHVWVGEIMRDVVAAPGDPVFFFHHTFIDYGWELFRQKINPNGNIDLR
241 NDYPNVGGFHAPDAPMFGFQGITNRDGYSDEYTRMYAPHPTCSNGCGGSTQFLYCPDGGP
301 MANPNRRCISREINSDLVPAAAVAPAAAMQAMTFGPEATMARFGPAATMGMSGPAATRAG
361 FSPAAVSMFGPAASRALFGPAAMTRAVPAVGSARVSVEATDAVAVRSALSEPPPPVSGIS
421 FESSFSDSRL
```

Figure S1

B

pfu_aug1.0_2323.1_15782.t1 (NU-5)

1 MTSQERRTKRSMTSYAKSALKLVTFKFTWEYILKYVKRVGLRQDKCREVVCVPGKILVDK

61 ECKPILQKTSGYGAYMDMSVTVLESKEGDYDLKEVFRDFAEAVMLRYNIEPIGDKTSTMN

121 IKEVVVLAYQPCTAQLTSDNTSEIYIQVTYESYASISREDVDRKLQEFVNPSFETRVYGF

181 LFDVKESDKNLWSILQSEQSEEGEYQIIDSRKITVRRFDEDHNISYTSEYGGVIRGAPVI

241 DVPNGYDFGGPPLPPPNFLQMRPPLLPFVGRDISARSPPSGSGVAITFGPSSVETGKSPG

301 GPKSNGPDKGYSVIDDAKSAPAGRRSINSRVLLQGGSGSRMMRERGPPSGANDKKNLICV

361 NGLGPGCGAPVLNGDSSSGKGKKIDDKGKLPDIGASGGEGNDPSNFCINGLGPGCGVEVI

421 DGNTGIPSGGKSGQNGIMDKNAAASDKKGTASKPGPTSNKNGFGPGPDMGGIDLGPDMGG

481 MGPGPNMGDMGSGPDVGGMGPGPDMGGMGPGPDMSGMEPAPDMGSIGPGPDMGGMGLGPE

541 IGGMGPGPDMGGMGPGPNNGGTGPGPDVGGMGTGPDMGGMGSGPDMGGIGQGPDMGGVGP

601 GAGMGGMGPGPSGSSSGAIGTAGIGLGGQMGIEVEIKSPEIEPRQAPEVKMPEIEMKQRP

661 EIKTPEIKIPEINTPEIKTPEIEPIQAIEIKVPEIEPQQGPEIEGPGFLPGSVGVPMANG

721 VGAGMQINGVSNVPGPSIDGGMSIDSGTLGMQANGVPDALAGSGLPVDSGSVGFKINGGP

781 NVQVPSKGVSVQMQSIGTGVLLNGGGGPSDNQYGGMVVGNGGMGFQINGGSDVPGASVGG

841 VNGNGGVVNNLQQMNGNINNQGSKSGMSGSSGASLPIFGSPVDTGSGGYNKMMSGSVDPP

901 GFLANGLDKPSDGNLGMLSPNKMNKPPSWLTDGFGDGSSGSGGGGGIDGGSGGSTGDNQI

961 KVSTGKVDGNKGSSSINIEVKKDAEVTLNIDQGGSKCICPPQITSTASPQMIKAGASIAK

1021 MSNKNVLIAKIPTAKLGGNTMFPNVEIPNLVIEPVSDNSYSSSSPGMTSSEFSLDSSGAS

1081 SDVLVVAALPGDLGTSGKVNVKVATDPMTGNLVIETTPSPMTGKGLGSSGAGSSSQGSSL

1141 GSVGCINGLGPNCGVPIVDGSTTGSXGPSSDASFGVPAPALAAAGLIPGQGAGGPATGKS

1201 AGAGPGKNVGGPEPGTSPVGAGPGTGPGVLGSSIDLGAMGPSLGGLGPAVSDGISSGGLG

1261 PSIDLGVIEPSSSVSGLGPAVPDGISSGALGTLEGNLGMPSVIGLDGTGLSLDIGSSPGV

1321 GGFDPSVGTSGTGNAAVSAGAGLVVDGTGFVIKKSDEDENKKLKVKCINGIGPDCVGITG

1381 GSSAGNVDSSVSVGGSGGITLAGVGEVKGMVDLASVGVDAGLNVGIGVADGSKIGDGLSG

1441 VDLNVDLGKVSGCVNGLGPGCGVPIIQGDTGAPVSVSVGGSSGPDAGIGGIVAKIKKEED

1501 GDKSGKISLVFASGNASAIDNMLSSTSLGLGMQQQTSIINAADPSIQQPSIGSSADLLSP

1561 LSEPGKSDLTILSKPSTSKSIGSKLLKKEEDGDGSGTSSSKGVLCINGLGPGCGVPIVSG

1621 SGDTLSSQPGSSLSGTGAQSPAPVPGGQAPSAPGSADPGLMAPSKSQGLPADILGLLGGG

1681 PSVPGITGPSSSSGGAGTDTSSGGPGTSGPSIGGSMGGGPAPSSSAGGVGSGTSVGAPGS

1741 GSKAGAPGSGGSSSKSPGTKGKSSASSSSPTSGGSPKPKTSSGSPGVSPLATALSKPAPK

1801 AKKPNLADRCLVPGVLKYSRSGGCNSYFKCIEGISVPACCPKGYKYDEDEECKPVSGIAD

1861 DVCDDDCDTPGDLDTLTSSSARKD

Supplementary Figure S1. Amino acid sequences of the protein identified from the nacreous microstructure. (A) shows the amino acid sequence of pfu_aug1.0_3212.1_37533.t1 (tyrosinase). (B) shows the amino acid sequence of pfu_aug1.0_2323.1_15782.t1 (NU-5).
Red color indicates peptide fragments detected by LC-MS/MS. A solid line in (A) indicates tyrosinase domain. A solid line in (B) indicates methionine-rich region. A dotted line in (B) indicates glutamic acid-rich region. A thick line in (B) indicates serine-rich region.

Figure S2

A

pfu_aug1.0_14699.1_32469.t1 (P,NU-5)

1 MQTAGSMRISHQVGGSMTGKALIGGSMGSKRMMSMSSSHSSHGSKLSGHGSGGSSYIIRK

61 VTKTSSTGHGLGGSTIIRKTVTTHHIKTTGKSGGMHKSSSSIVRHQSSSSGQTGVHNLGG

121 GAVVVHIKGSSSSGGQTGGVYKVAGSQSSMKSNKATMASGKASAVAMGSAALHNFIKQAS

181 TVYNSENKGEMKSALESNQESAAIRKFLLSNAGQGKGMTIGKESLSLSGQNNQKQAISVS

241 QVGQSTGQGQESGSAALQNFVTSTNEGETSVASAVNAQKDMASGTSTVTGGSSANLEIRN

301 TKSQNDNSETLSSSAITDSGSLKSNAAASLTGNAGGDIGESDQDSVNDGIIGADNNDGGD

361 SDDNKSGDNDDDNSSDNDDGNSGDNDDNNSGDNDDDNSGDNDVNSSDNDDDNRRWTTIMT

421 TMVMTRMMMIQLRREMQTLIMMEIKMIVIQQMMTVMIAPKIAISIQVSHLMMT

Figure S2

B

pfu_aug1.0_3525.1_23341.t1

1 MDIGNMSHTYALCRLELLKKVSREGFDKPVTMENAISSSSLNIVIEYVKEKKHMFPTRTT

61 IEDPFKQWLVNGGQCGVCGDPWDELRTNEILEQFPEIFMVDNIAKQKRIKLFAELPSNGR

121 GYIEVRLCESKTNQLSQECFDENLLFIEEANANRYKVGKETQVVLTLLVPETIICEKCVL

181 QWKYVKVPSNVQPPQANNNVAQNSMHLMTQQVNNNMNQNAPTPQSNYMGITDITKVLKPS

241 AAPYTGGNPPQQQTSNQNTQQTSRQGSSMIIQSQTQASSSTQGQVNQAHSNTMHSASHTS

301 QHQAINQQQMTNQQQVMNQQQMTNQQQVMNQQQGMNQQQMTNQQQVMNQQQMTNQQQVMN

361 QQQMTNQQQMTNQQQVMNQQQMMNQQQMMNQQQMTNQQQVMHQQQGMNQHQGMNQHQGMN

421 QQSTSNIHTAIKHHGTNKHTPSLLENMENSLKVIQSMDGKERNALYVVKSSKDVPKVIQT

481 LEMNKTKKTKEEVVAPVEQATPAEQYPGGNENMLNIKYDTGFSGGRNIPLYVVQTEPKQV

541 QATNTLPQTMNNLPASQNVLSKTVSKSSMIPPNPPPPKQVSPTLTEPSNVQNQNVISNTV

601 VNTQKSNILYDKPVEPIPKGVKLAGIPDPISSNIVIPKASQVSVAQNKYPTVQPSPKSQV

661 IAGSSSVGMEMKGPTQMTSLDAILWGVNAGKQATGTSMPLPKQPFPVSSFSTTNNNPKSE

721 VKFSARRPVFKPGKKAVFSFGYRNKQRTFSGKEPSVFKMPRAQDNAAQNQINQLEPKTAM

781 KSIESFNTANKDLMSSQDPRFGSSSSSMVSNAVMNMPNVNMMPPQAQVVNRPPSSFMNNA

841 GMTGAVVMTGFQGTGGRSTMTGNAQTEAAGVNMVSSGLSQSQSKQVQVLTSSLSASGQYS

901 MQDKQEQAMMGGNINPPNLLQSSVIVPEKPSMPADGRVDQAGMMVSEPMMNQMSSGSSAH

961 LSSSNTVGRQTHKFFDGNPEIVVSHSQGQSSQSNPVHKSKVLWSSAVSKNQGPNIIYRDQ

1021 SNPPTNPTMTPGVTQSKSPSYPSFSFTGIQSTAMATKVPLFNAAGTAGPGLLETKDYFSR

1081 TPIQKVINMRRPSFSFGSKSRNHSPKISYIKSFSVQQNQKQTNAPLNMTPTGNTMKPTTG

1141 QWNRVR

Supplementary Figure S2. Amino acid sequences of the protein identified from the prismatic microstructure. (A) shows the amino acid sequence of pfu_aug1.0_14699.1_32469.t1 (P,NU-5). (B) shows the amino acid sequence of pfu_aug1.0_3525.1_23341.t1.
Red color indicates peptide fragments detected by LC-MS/MS. A thick line in (A) indicates pnk superfamily domain. A solid line in (A) and (B) indicates methionine-rich region. A dotted line in (A) indicates aspartic-rich region. A dotted line in (B) indicates glutamic acid-rich region.

Figure S3

A

pfu_aug1.0_3952.1_44831.t1 (LMP)

```
1   MKFLAAVAFVAVLSHSTVYSYGPRNRGKMMPMMPPAKKMMNNDMMKMMKPDDAMMKPDKQ
61  NQMKPDDMKQMMKMMMDKMMMMDKMMMMDMMMKMMKPDMMMKPDMMMKPDMMMKPDMMMK
121 PDMMMKPDMMMKPDMMMKPDMMMKPDMMMKPDMMMKPDMMMKPDMMMKPDMMMKPDMMMK
181 PDMMMKPDMMMKPDMMMKPDMMMKPDMMMKPDMMMKPDMMMKPDMMMKPDMMMKPDMMPD
241 MMMKPDMMMKPDMMMKPDMMMKPDMMMKPDMMMKPDMGMMKPDMGMMMHPTPYNLYQG
```

Figure S3

B

pfu_aug1.0_1662.1_66071.t1

1 MDKPGNGRDGNGINGDKNNNGNSGSETPWDGQNGNNGNGNNNGHSGQYSPWVNMNGNNSG

61 SKTPWDSQNGNGNSNGNNGQYSPWISPNGNGNNNGNSGSKTPWDSQNGNGNSNGNNGQYS

121 PWISPNGNGNNNGIESSWDSQNGNGNNNGIKNPWDSQNGNGNSNGNSGKYSPWISPNGNG

181 NNNGNSDSKTPWYSQNGNGNSNGNNGQYSPWISPNGNGNNNGNSGSMTPWDSQNGNGNNN

241 GNGGSKSWWDSQNGNGNSGSKTLWDSQNGNGNSGSKTWWDSQNGNANNNGNNGQYGNNNG

301 NSGSETPWNSQNGNGNNNGNSGHKTLWHSPNANGMNSMQMATMDNTNDMKNMIDKMAMKM

361 MNDIKMINKMNMMNDMKIMNEIKMMNDMNMINAMKIMNKKDSTMNDQNSMDMINGGNNMM

421 NGMNGMDKMNGKMDMGAMNNGGNKMMNGMDGNTGKGSIMMMKMKDPKSKQPASIY

Supplementary Figure S3. Amino acid sequences of the protein identified from ligament. (A) shows the amino acid sequence of pfu_aug1.0_3952.1_44831.t1 (LMP). (B) shows the amino acid sequence of pfu_aug1.0_1662.1_66071.t1.
Red color indicates peptide fragments detected by LC-MS/MS. A solid line in (A) and (B) indicates methionine-rich region.

Figure S4

A

1 MGSSHHHHHHSSGLVPRGSHMMDKNAAASDKKGTASKPGPTSNKNGFGPGPDMGGIDLGPD
61 MGGMGPGPNMGDMGSGPDVGGMGPGPDMGGMGPGPDMSGMEPAPDMGSIGPGPDMGGMGLG
121 PEIGGMGPGPDMGGMGPGPNNGGTGPGPDVGGMGTGPDMGGMGSGPDMGGIGQGPDMGGVG
181 PGAGMGGMGPGPSGSSSGAIGTAGIGLGGQMGIEVEIKSPEIEPRQAPEVKMPEIEMKQRP
241 EIKTPEIKIPEINTPEIKTPEIEPIQAIEIKVPEIEPQQGPEIEGPGFLPGSVGVPMANGV
301 GAGMQINGVSNVPGPSIDGGMANGVPDALAGSGLPEF

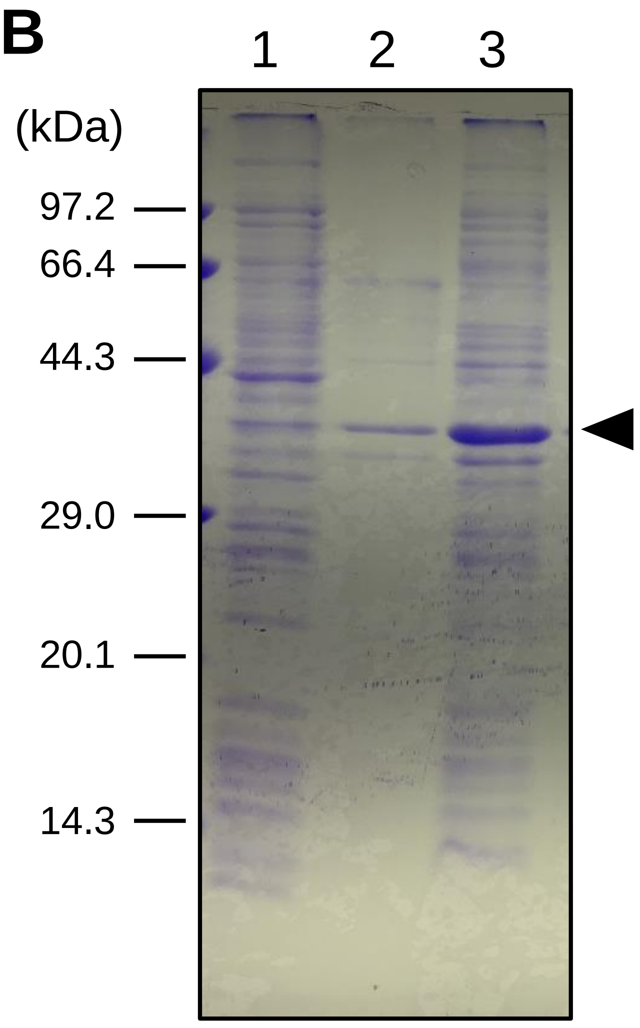

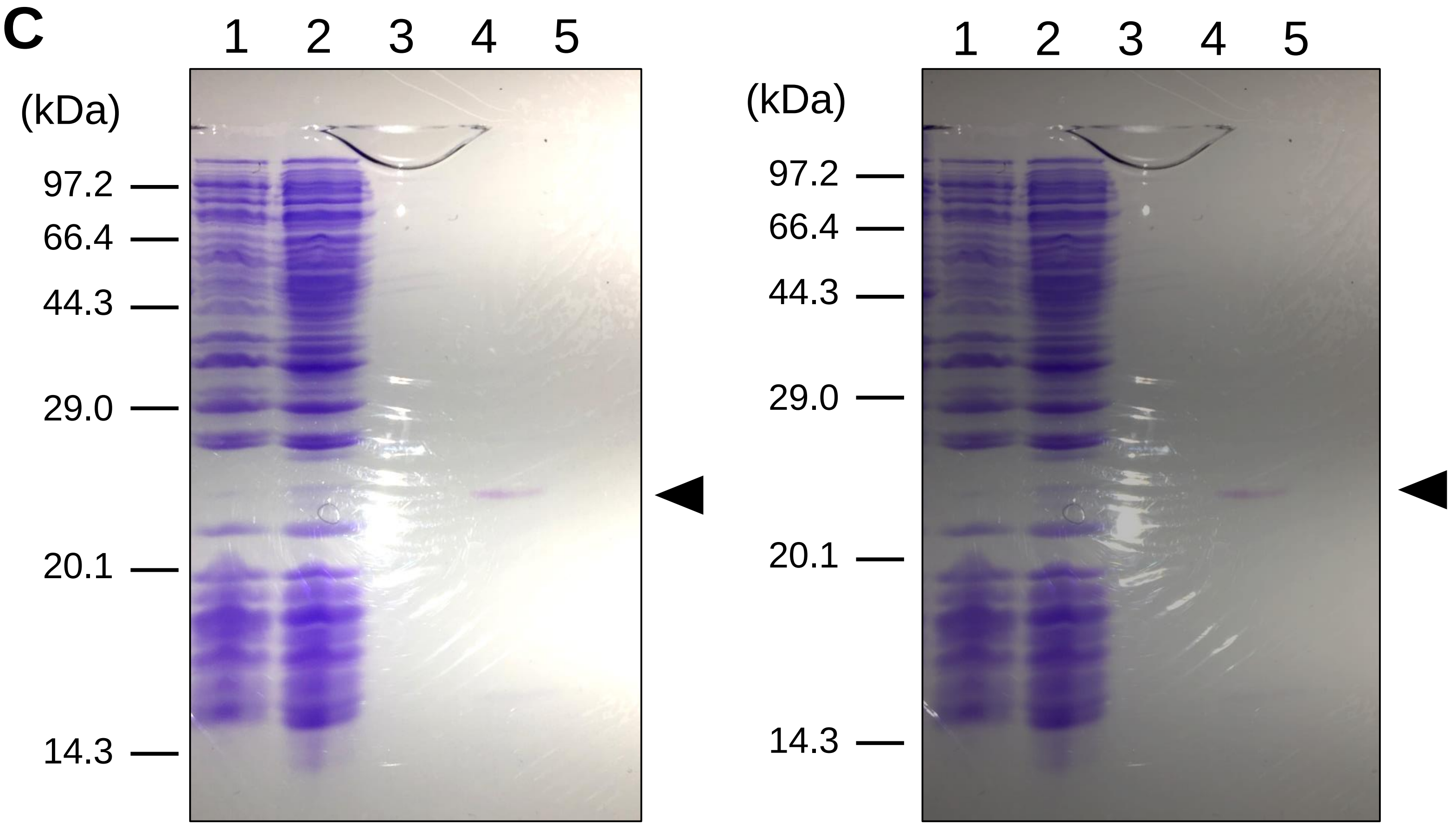

Supplementary Figure S4. (A) Amino acid sequence of rMERP. A solid line indicate His-tag. (B) A band pattern of the soluble fraction after expression of rMERP by *E. coli*. Lane 1 : Extract from *E. coli* with non-inserted vector, Lane 2, 3 : Extract from *E. coli* with rMERP-inserted vector made expressed by 0.1 mM IPTG (Lane : 2) and 0.5 mM IPTG (Lane : 3). An arrow head indicates the band of rMERP. (C) A band pattern during purification process by Ni column. Left gel showed the high brightness and contrast image. Right gel showed the low brightness and contrast image. Lane1 : Extract before purification, Lane 2~5 : Extract eluted through column by imidazole at concentration of 20 mM (Lane 2), 100 mM (Lane 3), 250 mM (Lane 4) and 500 mM (Lane 5). An arrow head indicates the band of rMERP.

Figure S5

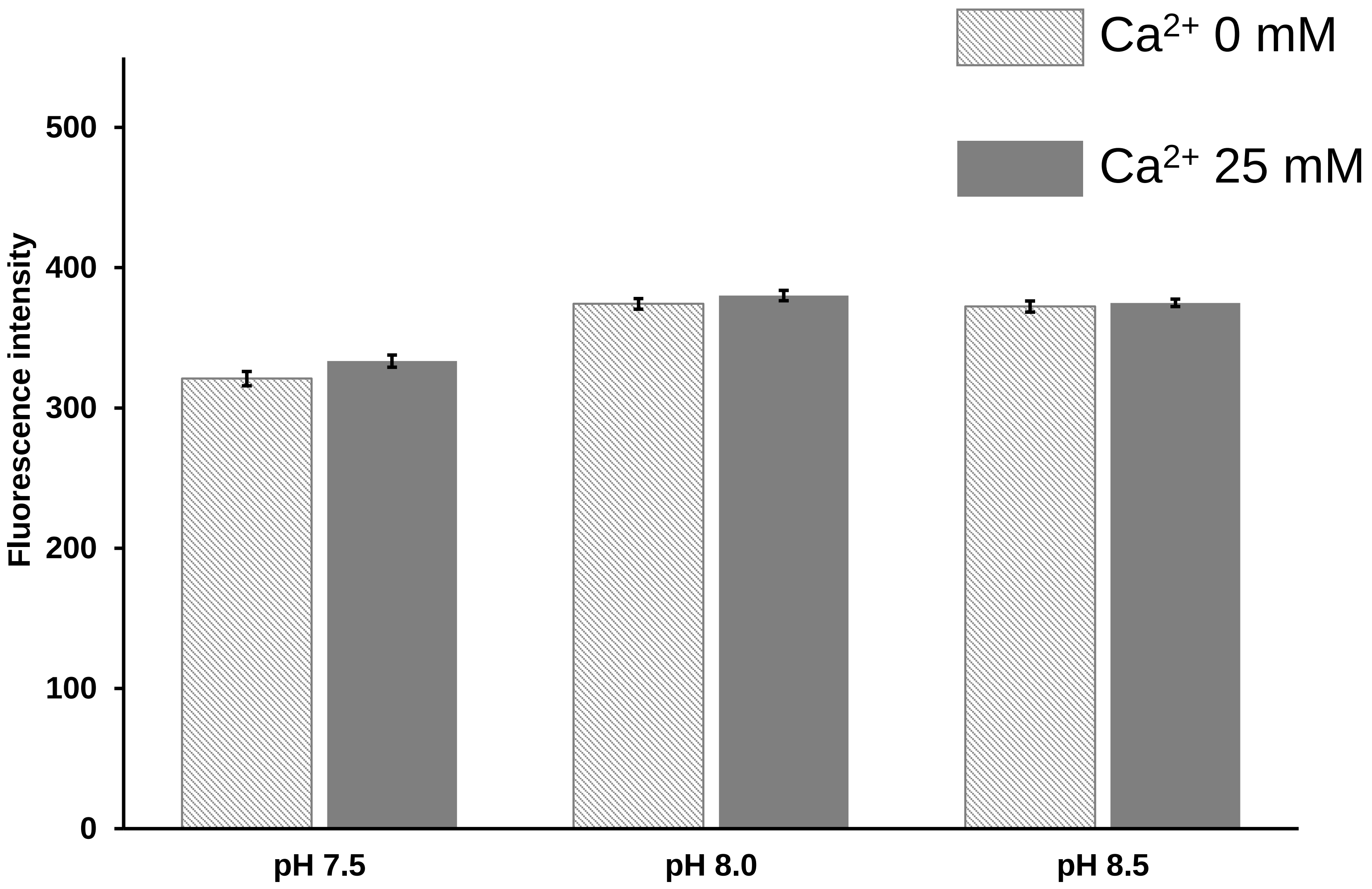

Supplementary Figure S5. Nile red staining. A steric structural change in the absence of presence of calcium ion at different pH was measured. Data are expressed as the mean ± S.E (*n* = 3).

Figure S6

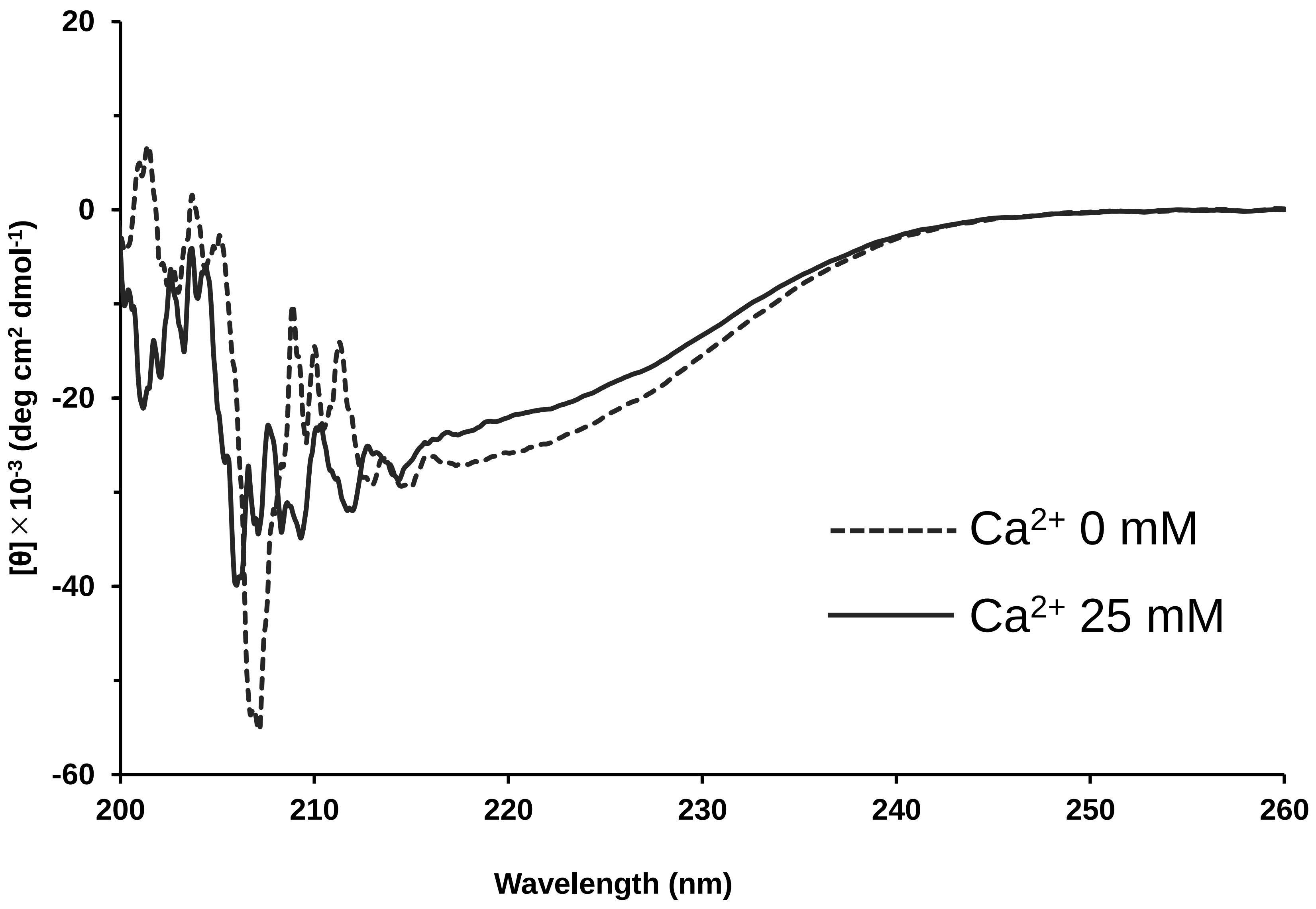

Supplementary Figure S6. Circular dichroism (CD) spectrum of rMERP. Secondary structures of rMERP in the absence or presence of calcium ion at pH 8.0 were analyzed.
